# EEG and IMU Gait Signal Processing: A Comparative Assessment of the “Reza” Exponential Filter and Classical Filters

**DOI:** 10.3390/s26051719

**Published:** 2026-03-09

**Authors:** Reza Pousti, Daniel M. Russell, Derek C. Monroe, Christopher K. Rhea

**Affiliations:** 1 Ellmer College of Health Sciences, Old Dominion University, Norfolk, VA 23529, USA; gpous001@odu.edu; 2 School of Exercise Science, Old Dominion University, Norfolk, VA 23529, USA; dmrussel@odu.edu; 3 Department of Exercise and Sport Science, University of North Carolina at Chapel Hill, Chapel Hill, NC 27599, USA; dcmonroe@unc.edu

**Keywords:** EEG filtering, IMU gait analysis, IIR filters (Butterworth, Chebyshev, elliptic), Reza filter, signal-to-noise ratio, power spectral density

## Abstract

Noise degrades both EEG and gait signals, and classical IIR filters (Butterworth, Chebyshev, elliptic) involve trade-offs between passband flatness, ripple, and roll-off. This study compared a novel exponential “Reza” filter with these designs for neural and locomotor data. We analyzed an open-source mobile brain–body imaging dataset with EEG and gait data from 49 healthy adults (EEG: 256-channel, 512 Hz; IMUs: six APDM Opals, 128 Hz). EEG channels were grand-averaged and band-pass filtered at 0.5–50 Hz, while IMU axes were averaged and band-pass filtered at 0.5–5 Hz. The outcomes were signal-to-noise ratio SNR (dB) and band-integrated Welch PSD (EEG:0.5–50 Hz; IMU:0.5–5 Hz). Repeated-measures ANOVAs tested the effect of filter types (Butterworth, Chebyshev I, elliptic, Reza) with Bonferroni-adjusted post hoc tests for the six pairwise filter comparisons ($\alpha_{adj}\;=\;0.0083$). We reported partial eta-squared ($\eta p^{2}$) as the ANOVA effect size. For EEG, PSD did not differ among filters (p = 0.146), whereas SNR differed strongly (p<0.001): Chebyshev and elliptic yielded the highest mean SNR and did not differ from each other, while both exceeded Butterworth, Reza was the lowest. For IMU, both SNR (p< 0.001) and PSD (p< 0.001) differed: Reza produced the highest mean SNR (significantly exceeding elliptic and Chebyshev), while Butterworth exceeded Chebyshev; meanwhile, IMU PSD showed a clear ordering with Reza retaining the most motion-band power, followed by Butterworth, then Chebyshev, with elliptic retaining the least. These results showed that filter choice materially shapes EEG and gait outcomes. For EEG, Chebyshev maximized SNR, while elliptic and Reza maintained comparable fidelity. For IMU gait signals, Reza matched Butterworth for denoising and preserved more signal power. Therefore, filter choice should be guided by the target outcome (SNR vs. band power) rather than a single default design.

## 1. Introduction

Noise is a constant adversary in physiological measurement. During data acquisition, physiological signals are corrupted by thermal and electronic noise, baseline drifts, and motion-induced artifacts, and the measuring devices themselves can introduce noise through sensor limitations and amplifier circuitry [1,2,3,4]. Physiological noise sources such as cardiac pulsation, respiration, and low-frequency vasomotor oscillations generate time-varying signals that can obscure subtle notable changes in brain imaging data [5,6]. In electroencephalography (EEG), this vulnerability is amplified: the high temporal resolution of EEG means that scalp potentials are readily contaminated by ocular activity, cranial and facial muscle contractions, line noise, and head motion, giving rise to a wide range of artifacts [7,8,9]. Biomechanical motion data can face similar challenges. To suppress high-frequency noise and stabilize numerical differentiation when computing velocities and accelerations, marker trajectories and inertial signals are typically low-pass filtered with cut-off frequencies between 3 and 10 Hz, though most commonly around 6 Hz for level walking [10,11,12]. Walking while wearing mobile EEG can add yet another layer of complexity where large-scale body movements, cable sway, electrode–skin impedance changes, and gait-synchronized head motion all contribute movement-related artifacts that can confound or even swamp the underlying brain activity patterns, complicating the interpretation of mobile brain–body imaging data [13,14,15,16,17].

Because noise is unavoidable, filtering is non-negotiable. However, filters inevitably alter the data they process beyond attenuating unwanted frequencies—filters can also impose undesirable frequency-dependent phase shifts that introduce time lags and can disrupt timing relationships within and between signals. Filter-based phase shifts have been shown to distort neural synchrony and cross-frequency coupling, potentially leading to misinterpretation of neural timing and connectivity measures [18,19]. Commonly used infinite impulse response (IIR) notch and band-pass filters, such as Butterworth designs, can exhibit nonlinear phase responses; when such filters are applied forward and backward to achieve a nominally zero-phase response, the resulting acausal filtering can smear event-related potentials, distort waveform morphology, and shift apparent onset latencies, in some cases even creating artifactual deflections or oscillations [20,21,22,23]. Thus, the central goal of signal processing is to suppress noise as aggressively as necessary while minimizing damage to the underlying physiological signal.

**Figure 1:**
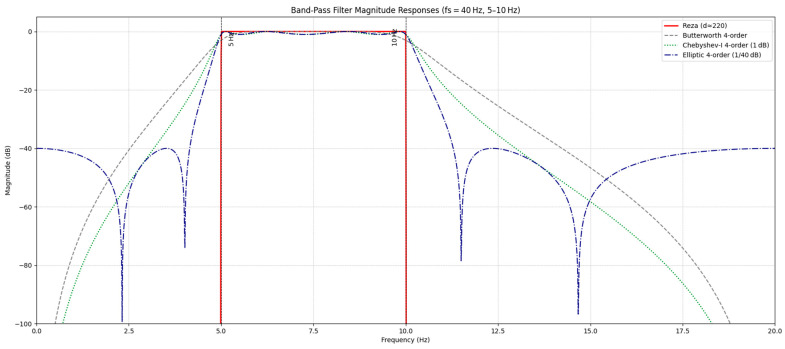
Illustrative magnitude responses of four digital band-pass filters on simulated data (fs = 40 Hz; pass band = 5–10 Hz) under a specified parameter set. This example is shown to compare canonical frequency response shapes; empirical performance in the EEG/IMU analyses depends on sampling rate, band edges, record length, and boundary handling.

Digital filtering nevertheless remains ubiquitous in practice. In biomechanical analysis, zero-phase Butterworth low-pass filters are among the most widely used, where kinematic data are frequently filtered forward and backward with low cut-off frequencies (e.g., 3–10 Hz for gait) to improve signal-to-noise ratio and stabilize numerical differentiation [10,11,12,24]. In EEG preprocessing, Butterworth filters implemented as notch, high-pass, or low-pass stages are also common, despite clear demonstrations that suboptimal choices of filter type and cut-off can introduce temporal smearing, spurious peaks, and biased estimates of neural activity [20,22,23]. Yet classical IIR designs carry characteristic trade-offs. Butterworth filters can adopt a maximally flat magnitude approximation in the passband with a monotonic stopband, but this smoothness comes at the cost of a relatively gradual roll-off, so higher orders are required to achieve strong attenuation at distant frequencies [25,26,27]. Chebyshev Type I filters can sharpen the transition band by allowing equiripple gain in the passband, producing a substantially narrower transition region than an equal-order Butterworth design, but the passband ripple and associated group-delay ripple can introduce amplitude distortion and more nonlinear phase [28,29,30]. Elliptic (Cauer) filters go further, permitting ripple in both passband and stopband to achieve the steepest roll-off and narrowest transition width for a given order, but they can exhibit highly nonlinear phase and more irregular magnitude responses than Butterworth designs [28,31,32]. EEG preprocessing choices directly shape downstream decoding, as artifact suppression and filter design can influence feature fidelity, affective BCI robustness, and cross-subject transfer, including privacy-preserving schemes relying on stable predictions. Aggressive/steep filters can introduce ringing and temporal distortions, requiring impulse response/settling time evaluations [33,34,35,36]. These ripple and phase nonlinearities can warp waveform timing and morphology, forcing a continual calculus of compromises between steep attenuation and faithful preservation of physiological signals; accordingly, we summarized the corresponding magnitude and phase characteristics of the candidate filter designs in Figure 1 to provide a visual reference for readers.

To reduce these trade-offs while retaining tunable control over attenuation, we have explored an alternative window-based approach. The Reza filter is an exponential window-based design that replaces polynomial window approximations (such as the Bessel-series form of the Kaiser window) with an exponentially shaped kernel, reducing computational complexity while preserving control over main lobe width and ripple [37,38]. Prior work on exponential windows has shown that, for a fixed window length and main-lobe width, exponential windows can achieve a higher sidelobe roll-off ratio than the classical Kaiser window, meaning that sidelobe energy decays more rapidly away from the main lobe [37,38,39]. Collectively, these spectral properties allow exponential window filters to realize steep effective transition bands for a given filter order while maintaining a relatively smooth passband and tunable ripple, and they have already been exploited in biomedical applications such as electrocardiogram sub-band processing [38,40]. Such characteristics have made exponential window filters attractive for biosignals that demand both ruthless noise rejection and faithful temporal detail. To test this premise in a realistic mobile brain–body context, we compared a Reza filter with Butterworth, Chebyshev, and elliptic filters using the open mobile brain–body dataset of indoor treadmill and outdoor overground walking from Hanada, Kalabic, and Ferris (2024) [41]. This work provided a focused, reproducible benchmark for how commonly used band-pass filters can shape EEG and IMU gait signal quality. Specifically, we (1) formalized the Reza filter and its parameterization for practical biosignal band-pass filtering; (2) compared Reza against Butterworth, Chebyshev Type I, and elliptic designs on an open EEG–IMU gait dataset comprising high-density EEG and multi-sensor IMU recordings; (3) quantified the impact of filter choice on residual-based signal-to-noise ratio and band-integrated Welch power spectral density using repeated-measures statistics with effect sizes; and (4) provided transparent parameter settings and open-source implementations to support independent replication and downstream testing. It was hypothesized that the Reza filter would lead to greater signal-to-noise ratio and greater broadband power spectral density (i.e., higher total band power across the analyzed frequency range) relative to the traditional filters.

## 2. Materials and Methods

### 2.1. Dataset and Instrumentation

We analyzed the open-access mobile brain–body signal dataset of 49 young, healthy adults who performed a visual search task outdoors on a natural arboretum path [41]. In the outdoor session, subjects walked continuously for approximately one hour while completing a 20 min under a baseline walking condition (no flags), followed by two 20 min under visual search conditions (non-stress and induced-stress) in randomized order where participants pressed a handheld joystick button when they detected the target flags. EEG was recorded with a 256-channel BioSemi ActiveTwo system (Amsterdam, The Netherlands) at 512 Hz, with eight additional neck electromyography sensors. Participants also wore six APDM Opal (Portland, OR, USA) inertial measurement units (IMUs) at 128 Hz attached to both feet, both ankles, the waist, and the chest. EEG and IMU streams were synchronized to EEG using LabVIEW triggers and confirmed to be aligned within 1–2 data frames of each device’s sampling rate. Although the source dataset contains 49 participants, differences in sensor configuration and occasional database/file errors meant that not all individuals contributed usable data to every analysis $\left(n=47\;for\;EEG\;ANOVAs\;and\;n\;=\;46\;for\;IMU\;ANOVAs\right)$. The full dataset is openly available on IEEE Dataport (DOI:10.21227/H24T0V) and Figshare (DOI:10.6084/m9.figshare.6741734).

### 2.2. EEG Preprocessing

EEG was recorded from 256 scalp channels. For the present analyses, as regional topography is not the primary focus, we reduced the high-dimensional sensor space to a single global trace per participant by averaging the voltage across all channels at each time point, yielding a simple surrogate of whole-head cortical activity. The resulting grand-average signal was band-pass filtered from 0.5 to 50 Hz using a zero-phase digital filter, as this range spanned the canonical delta–through–low-gamma bands and has been widely used to retain behaviorally relevant EEG activity while suppressing slow drifts and high-frequency noise in contemporary EEG and event-related potential (ERP) pipelines [42,43,44].

### 2.3. IMU Preprocessing

Tri-axial accelerometer (X, Y, Z) and gyroscope (X, Y, Z) signals were likewise collapsed to a single composite six-degree-of-freedom trace per participant by averaging each axis across sensors analogous to vector-magnitude summaries commonly used in gait accelerometry [45,46]. The composite time series was then band-pass filtered between 0.5 and 5 Hz. This range approximately covered the dominant frequency content of human walking and running (∼0.5–5 Hz) and has been used to isolate gait-related oscillations and gait phase dynamics while attenuating sensor chatter, tremor, and other non-gait movements [47,48]. This preprocessing therefore preserved stride-to-stride content while removing very slow drift and higher-frequency noise.

### 2.4. Filter Designs

#### 2.4.1. Reza Filter Parameters

The Reza filter (Equation (1)) was designed to achieve a steep cutoff with minimal ripple and phase distortion by explicitly shaping its ideal magnitude response with an exponential function. In the frequency domain, the target gain profile is:

(1) $$H\left(f\right)=exp\left(-c\cdot {\left(\left|f-f_{c}\right|+offset\right)}^{d}\right)$$

where

- f is the frequency;
- $f_{c}\;$ is the cutoff frequency (or the central frequency for band-pass);
- c is a coefficient determining the initial rate of attenuation;
- offset is a small value to ensure proper behavior at the cutoff frequency;
- d controls the exponential rate of decay, affecting the sharpness of the cutoff.

By tuning $\left(c,\;offset,\;d\right)$, the Reza filter can approximate a “razor-sharp” transition while maintaining a smooth, monotonic passband and a controlled, nearly symmetric attenuation profile around $f_{c}$, in contrast to the polynomial magnitude approximations used in classical IIR and window-based finite impulse response designs. The exponent d, which governs the steepness of the exponential roll-off, was determined automatically from the data rather than fixed a priori. For each dataset, and for a given sampling rate $f_{s}$, data length N, and band-pass edges $\left(f_{c1},f_{c2}\right)$, we first computed the one-sided FFT frequency grid $f_{k}$ and the corresponding Reza band-pass gain $G_{d}\left(f_{k}\right)$ for an initial value $d_{0}=10$. Edge sharpness (Equation (2)) was quantified as the average gain jump across the lower and upper-band edges:

(2) $$S\left(d\right)=\frac{1}{2}\left(\left|G_{d}\left(f_{c1}\right)-G_{d}\left(f_{c1-∆f}\right)\right|+\left|G_{d}\left(f_{c2}\right)-G_{d}\left(f_{c2+∆f}\right)\right|\right),$$

where ∆f is one FFT bin, and $G_{d}\left(f_{c1\pm ∆f}\right),\;G_{d}\left(f_{c2\pm ∆f}\right)$ are evaluated at the nearest discrete frequencies below/above $f_{c1}$ and $f_{c2}$. Starting from $d_{0}$, the algorithm increased d in fixed steps (Δd=5) and recomputed S(d) until the change in sharpness between successive iterations fell below a small tolerance $\left(\left|S\left(d\right)-S\left(d_{prev}\right)\right|<10^{-4}\right)$. The converged value of d was then used to construct the final exponential band-pass template and frequency domain gain for that dataset. This adaptive procedure was applied identically for IMU (0.5–5 Hz) and EEG (0.5–50 Hz) filtering. Because the Reza filter was implemented via real-valued magnitude weighting in the frequency domain, it preserved phase (zero-phase) and was therefore an offline, noncausal filtering approach in the present benchmark.

The full Reza-Filter repository (including high-pass, low-pass, and band-pass implementations, example scripts, and analysis utilities) is available at in the Supplementary Materials and via the following link:

https://github.com/Rezapousti/Reza-Filter.git, accessed on 9 March 2026

For Python users, the package can be installed via pip:

pip install reza-filter

After installation, it can be imported and used as:

import reza

For MATLAB users, the Reza-Filter toolbox is available on MATLAB File Exchange and can be installed as a standard .mltbx add-on. After installation, the primary API can be called directly from the MATLAB command line, e.g., y = rezafilt(x, Fs, [5 50], ‘bandpass’, ‘UseMex’,’auto’).

#### 2.4.2. Butterworth

##### EEG Configuration

For EEG, we used a fourth-order Butterworth band-pass filter with a passband of 0.5–50 Hz. The sampling rate $f_{s}$ for each recording was read from the EEG file header using MNE-Python. Filter coefficients (b,a) were designed in Python with the SciPy signal toolbox and applied to the channel-averaged EEG time series using a forward–backward (two-pass) zero-phase implementation (filtfilt), which eliminated phase delay by filtering the data in both directions. This approach for a Butterworth IIR design with acausal, zero-phase application was consistent with current recommendations for EEG/ERP preprocessing when phase distortions must be minimized. For prospective real-time or online pipelines, we would recommend implementing the same fourth-order Butterworth band-pass in second-order sections (SOS) form (sosfilt) to improve numerical stability at low frequencies while maintaining the same magnitude response.

##### IMU Configuration

For IMU data, we used a fourth-order Butterworth band-pass filter with a passband of 0.5–5 Hz to isolate the gait fundamental and its lower harmonics. The sampling rate $f_{s}$ was read from the IMU .mat structure (field samplingRate). Filter coefficients were again obtained with scipy.signal.butter (order =4, band-pass) and applied using filtfilt to achieve zero-phase attenuation of out-of-band energy following common practice in biomechanical signal processing. For multi-channel IMU arrays, tri-axial accelerometer and gyroscope signals were first averaged across channels in the time domain to obtain a single representative six-degree-of-freedom trace per participant, which was then filtered. The choice of Butterworth filtering with forward–reverse application was consistent with established methods for smoothing gait kinematics and kinetics and with comparative work on Butterworth characteristics in biomechanical contexts.

#### 2.4.3. Chebyshev

Classical Chebyshev Type I filters can achieve a steeper transition band than equal-order Butterworth designs by allowing controlled ripple in the passband at the cost of greater passband distortion and more nonlinear phase. We used these filters as a contrast case to probe how passband ripple and sharper roll-off affect EEG and IMU signals in mobile brain–body data

##### EEG Configuration

For EEG, we implemented a fourth-order Chebyshev Type I band-pass filter with 0.5 dB passband ripple (Rp) and a passband of 0.5–50 Hz. Filter coefficients were designed using a standard Chebyshev Type I prototype (cheby1) with normalized band-edges and realized directly in second-order sections (SOS) form. For the benchmark comparisons reported in this manuscript, Chebyshev Type I and elliptic band-pass filters were applied using forward–backward zero-phase filtering (i.e., zero-phase SOS filtering) to prevent phase handling from confounding SNR and PSD estimates. A causal SOS implementation was feasible for real-time deployment; however, causal filtering was not used to compute the empirical statistics reported here. Chebyshev Type I filters’ sharp transition band for a given order, driven by their equiripple passband approximation, has been well-documented in classical filter design references and modern handbooks, and their nonlinear phase behavior has been highlighted as a potential concern in electrophysiological applications.

##### IMU Configuration

For IMU signals, we used a fourth-order Chebyshev Type I band-pass filter with 1 dB passband ripple (Rp) and a passband of 0.5–5 Hz to target the gait-related frequency range. As above, coefficients were obtained via cheby1. For offline analyses, the filter was applied in a zero-phase forward–backward manner (filtfilt) to the composite IMU trace to remove group-delay and phase distortions that would otherwise bias stride-timing and waveform-based measurements. This combination of Chebyshev magnitude characteristics with acausal, zero-phase application allowed us to isolate the effects of the sharper transition and passband ripple on gait-related dynamics while preserving accurate timing in the offline benchmarks.

#### 2.4.4. Elliptic

Elliptic (Cauer) filters represent the most selective of the classical IIR approximations: for specified passband ripple and stopband attenuation, they can use the lowest possible order with equiripple behavior in both passband and stopband, and very steep transition bands [49].

##### EEG Configuration

For EEG, we implemented a fourth-order elliptic (Cauer) band-pass filter with 1 dB passband ripple $\left(R_{s}\right)$ and 40 dB minimum stopband attenuation $\left(R_{s}\right)$ over a passband of 0.5–50 Hz. Filter coefficients were designed using an elliptic prototype (ellip) with normalized band edges and realized directly in second-order sections (SOS) form. The filter was applied causally (sosfilt) to the channel-averaged EEG time series to model a realistic real-time implementation in which aggressive spectral selectivity is desired. Because elliptic filters can achieve their steep roll-off by permitting ripple in both passband and stopband, they provided a useful “worst-case” benchmark for examining how strong magnitude selectivity and nonlinear phase behavior can impact electrophysiological signals [49].

##### IMU Configuration

For IMU data, we used a fourth-order elliptic band-pass filter with 1 dB passband ripple and 40 dB stopband attenuation over a 0.5–5 Hz passband, targeting the gait-related frequency range. As above, coefficients were obtained from an elliptic design, and for offline analyses the filter was applied using a forward–backward, zero-phase procedure (filtfilt) to remove group-delay distortions while retaining the sharp magnitude characteristics. This configuration allowed us to exploit the high selectivity and minimal order of elliptic designs as an upper-bound comparison for noise rejection in gait signals, while still preserving accurate stride-timing information in the offline benchmarks.

### 2.5. Evaluation Metrics and Core Equations

We quantified filter performance using signal-to-noise ratio (SNR) and power spectral density (PSD)-based measures. SNR was expressed in decibels as a ratio of signal power to noise power by following standard definitions in digital signal processing (Equation (3)):

(3) $${SNR}_{dB}=10\;\log_{10}\left(\frac{P_{signal}}{P_{noise}}\right)$$

The theoretical PSD of a wide-sense stationary process x(t) was defined as the Fourier transform of its autocorrelation function $R_{xx}\;(\tau )$ (Equation (4)):

(4) $$S_{xx}\left(f\right)=\int_{-\infty }^{\infty }R_{xx}\left(\tau \right)e^{-j2\pi f\tau }d\tau$$

consistent with the Wiener–Khinchin theorem. In practice, $S_{xx}\left(f\right)$ was estimated using standard FFT-based methods (Welch-type averaged periodograms) which reduced variance by segmenting, windowing, and averaging spectra across overlapping segments.

For IMU signals, band-limited power in the gait band (0.5–5 Hz) was computed as (Equation (5)):

(5) $$P_{band}=\int_{0.5}^{5}S_{xx}\left(f\right)df\approx \sum_{k:\;0.5\;\leq \;f_{k}\;\leq \;5}S_{xx}\left(f_{k}\right)∆f$$

where $f_{k}$ are discrete frequency bins and ∆f is the frequency resolution. For EEG, band-limited power in the 0.5–50 Hz range was analogously defined as (Equation (6)):

(6) $$P_{band}=\int_{0.5}^{50}S_{xx}\left(f\right)df\approx \sum_{k:\;0.5\;\leq \;f_{k}\;\leq \;50}S_{xx}\left(f_{k}\right)∆f$$

Total spectral power $P_{total}$ for each trace was obtained by integrating the PSD over the full analyzed frequency range, which was consistent with Parseval’s relation between time domain variance and frequency domain power. PSD was estimated using Welch’s method which partitioned the continuous recording into short, partially overlapping segments (EEG: $n_{perseg}=1024$ samples ≈2.0 s at 512 Hz) and averaged the segment periodograms; therefore, $P_{total}$ and all band-power summaries reflected an average across epochs rather than assuming stationarity over the entire recording.

Band-limited SNR measures then contrasted power inside the band of interest with power outside that band. For IMU (Equation (7)),

(7) $${SNR}_{0.5-5}=10\log_{10}\left(\frac{P_{band}}{P_{total}-P_{band}}\right)$$

and for EEG (Equation (8)),

(8) $${SNR}_{0.5-50}=10\log_{10}\left(\frac{P_{band}}{P_{total}-P_{band}}\right)$$

### 2.6. Statistical Analysis

We ran four repeated-measures ANOVAs: SNR-EEG, PSD-EEG, SNR-IMU, and PSD-IMU, with each with filter type (Butterworth, Chebyshev-I, elliptic, Reza) as the lone factor. Alongside *p*-values, we reported partial eta-squared $\left(\eta p^{2}\right)$ as the ANOVA effect-size measure, which quantified the proportion of within-subject variance in the outcome attributable to the filter type after accounting for subject effects. Post hoc Bonferroni corrections ($\alpha_{adj}\;=\;0.0083$) preserved familywise error control.

### 2.7. Implementation Details

All analyses were performed in Python 3.x using NumPy for array operations and SciPy for signal processing routines (Butterworth, Chebyshev Type I, and elliptic designs; zero-phase and causal filtering; Welch spectral estimates). EEG data were imported from EEGLAB .set files using MNE-Python, an open-source toolbox for processing and analyzing electrophysiological data. Tabular outputs and metadata were handled with pandas, and visualization (where used) employed matplotlib. For interactive file and directory selection in batch tools, we used the standard tkinter graphical user interface.

Offline analyses used double-pass zero-phase filtering (filtfilt) for Butterworth-IMU, Chebyshev-IMU, and elliptic-IMU configurations to eliminate phase-delay and group-delay distortions in timing-sensitive measurements. For causal, low-latency pipelines, we implemented Chebyshev Type I and elliptic EEG filters in second-order sections form and applied them with sosfilt, which improved numerical stability at low frequencies while preserving the designed magnitude response.

All filter orders, passband and stopband edges, and ripple/attenuation targets (e.g., $R_{p}$, $R_{s}$) have been specified in Section 2 to enable independent re-implementation. The frequency bands used for EEG (0.5–50 Hz) and IMU (0.5–5 Hz) have been explicitly reported for each filter family.

Power spectral density was estimated using Welch’s method as implemented in SciPy’s signal.welch, which averaged periodograms of overlapping or non-overlapping windowed segments to reduce variance. We used SciPy’s default settings except where noted: Hann window, no overlap, detrend=“constant”, scaling=“density”, and segment length nperseg = 1024. This was consistent with standard practices for PSD estimation in physiological and biomedical signal analysis software and libraries: Python 3.x with NumPy, SciPy (signal: butter, cheby1, ellip, filtfilt, sosfilt, welch), MNE-Python (EEGLAB .set import), pandas and (optionally) matplotlib. For interactive file selection in our batch tools, we used tkinter (GUI).

## 3. Results

### 3.1. Frequency Response Comparison

Figure 1 shows the comparative magnitude responses of four digital band-pass filters on simulated data (fs = 40 Hz, pass−band = 5–10 Hz). The main panel shows attenuation (dB) versus frequency for the Reza (solid red), Butterworth (dashed gray), Chebyshev-I (dotted green), and elliptic (dash-dot navy) designs.

- **Reza filter:** The red trace sat flat at ≈0 dB across the entire 5–10 Hz passband, then plunged almost vertically on both sides, hitting ≈−100 dB by ~4.9 Hz and ~10.1 Hz. No ripple or transition ringing was detectable, confirming its “brick-wall” behavior in a band-pass setting.
- **Butterworth (fourth-order):** The gray curve exhibited the classic maximally flat profile inside the passband but showed the slowest roll-off of the group. Attenuation was only ≈−24 dB at 3 Hz and 12 Hz, illustrating the gentle 24 dB/octave slope typical of this prototype.
- **Chebyshev-I (fourth-order):** The green line traded ≈2 dB of ripple inside the 5–10 Hz plateau for a noticeably steeper skirt, delivering ~ 6–10 dB more rejection than Butterworth between 3–4 Hz and 11–12 Hz while still maintaining unity gain in-band, though it did introduce some distortion.
- **Elliptic (fourth-order):** The navy trace maximized selectivity by allowing ripple in both bands. Deep stopband notches reached below −70 dB around 2.5 Hz and 14.5 Hz, giving the best overall attenuation outside the passband, albeit with visible distortion ripple peaks and troughs.

### 3.2. EEG Results

**Figure 2:**
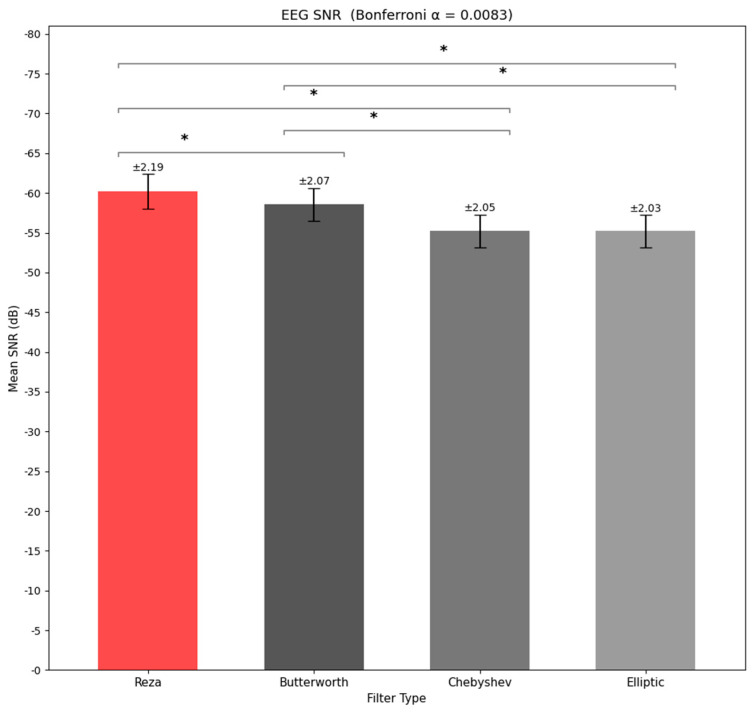
EEG SNR comparison across filters (The error bars represent ±1 Standard Error of the Mean (SEM) across participants). The (*) marks the specific post-hoc paired significant comparisons.

- **PSD Analysis:** A repeated-measures ANOVA on band-limited EEG power (0.5−50 Hz) revealed no significant effect of filter type $\left(F(3,135)=1.82,\;p=0.146,\;\eta p^{2}=0.039\right)$, indicating that, at this coarse spectral level, all four designs preserved the overall EEG power spectrum to a similar degree.
- **SNR Analysis (**Figure 2**):** In contrast, the filter type had a robust effect on EEG SNR $\left(F\left(3,135\right)=44.00,\;p=6.72\times 10^{-20},\eta p^{2}=0.494\right)$. Bonferroni-corrected pairwise comparisons $\left(\alpha_{adj}=0.0083\right)$ indicated that Reza produced a significantly lower (more negative) SNR than Butterworth, Chebyshev, and elliptic, while Chebyshev and elliptic did not differ from each other. Butterworth was intermediate but significantly lower than both Chebyshev and elliptic. Negative SNR values were plausible in scalp EEG because extracranial myogenic/ocular activity can mask neuronal activity, and an SNR expressed in dB would drop below 0 dB when noise power exceeded signal power [8,35,50].

### 3.3. IMU Results

**Figure 3:**
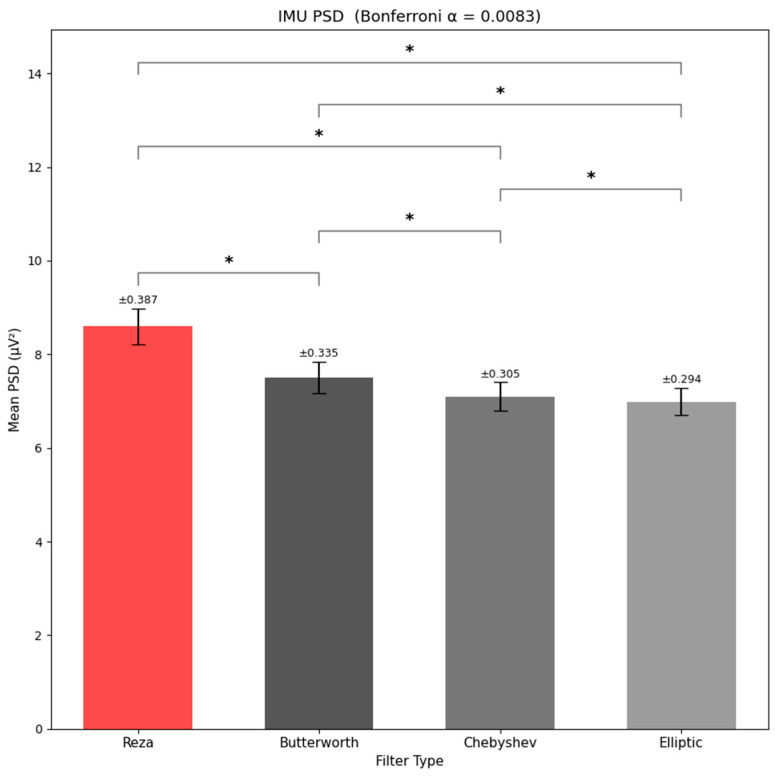
IMU PSD comparison across filters (The error bars represent ±1 Standard Error of the Mean (SEM) across participants). The (*) marks the specific post-hoc paired significant comparisons.

**Figure 4:**
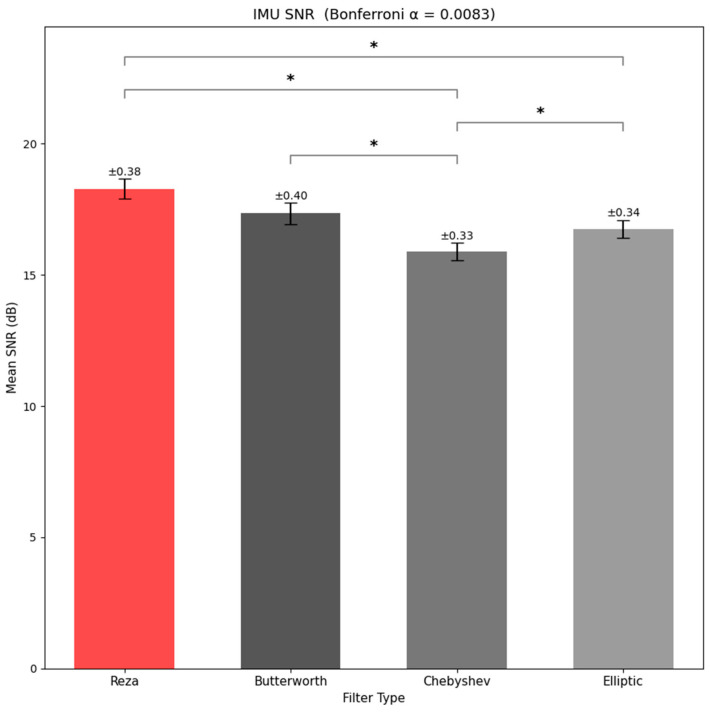
IMU SNR comparison across filters (The error bars represent ±1 Standard Error of the Mean (SEM) across participants). The (*) marks the specific post-hoc paired significant comparisons.

- **PSD Analysis (**Figure 3**)**: Band-limited IMU power (0.5–5 Hz) differed strongly by filter type $\left(F\left(3,126\right)=171.09,\;p=2.97\times 10^{-44},\eta p^{2}=0.803\right)$. Bonferroni-corrected pairwise comparisons $\left(\alpha_{adj}=0.0083\right)$ indicated that the Reza filter retained the highest motion-related power in the gait band, followed by Butterworth, with Chebyshev and elliptic retaining less (all pairwise differences significant).
- **SNR Analysis (**Figure 4**)**: The repeated-measures ANOVA on IMU SNR revealed a highly significant main effect of filter type $\left(F\left(3,126\right)=31.69,\;p=2.47\times 10^{-15},\eta p^{2}=0.430\right)$. Bonferroni-corrected pairwise comparisons $\left(\alpha_{adj}=0.0083\right)$ showed that Reza yielded higher SNR than Chebyshev and elliptic, while its difference versus Butterworth did not reach the adjusted significance threshold. Butterworth also exceeded Chebyshev, whereas Butterworth and elliptic did not differ significantly. Thus, for gait-band IMU data, Reza and Butterworth offered the most effective suppression of out-of-band noise while preserving the within-band signal. In combination with the PSD results, this pattern indicated that Reza and, to a slightly lesser extent, Butterworth achieved a favorable balance analyzing gait analysis (i.e., high SNR) without excessively shaving genuine stride-to-stride signal content.

## 4. Discussion

Although EEG total power in the 0.5–50 Hz band remained constant across designs, each filter imposed a distinct fingerprint on the waveform because different IIR prototypes can modulate amplitude and phase differently, which can shape the waveform and distort timing relationships [18,30]. Chebyshev Type I filters achieved their knife-edge roll-off by allowing equiripple gain in the passband and yielding a narrower transition region, but this also introduced amplitude modulations (passband ripple) and a nonlinear phase response [30,51]. Such ripples can nudge a μ-rhythm peak up or a beta burst down by around a decibel, and these small differences matter when tracking subtle cortical signatures [18,22,51]. Butterworth filters, in contrast, provided a maximally flat, monotonic passband with a gentle slope; this flatness preserved spectral peaks but left more residual noise, explaining their lower SNR in our data [28,30,51]. Elliptic filters permitted ripple in both passband and stopband to attain the steepest roll-off and the smallest transition width for a given order, but they exhibited highly nonlinear phase and irregular amplitude responses [30,51]. The Reza filter paired near-elliptic stopband attenuation with a smooth passband, as exponential window filters have been shown to yield higher sidelobe roll-off ratios and greater far-end stopband attenuation than classical Kaiser and Cosh windows [37,51]; therefore, our results showed that the Reza filter matched or surpassed the classical designs’ attenuation while avoiding passband ripple. Although the Reza filter used a configuration-adaptive exponent selection, this procedure was deterministic from the recording configuration (sampling rate, band edges, FFT grid) and was not tuned to maximize SNR or PSD, so it should not be interpreted as participant-level optimization. Filtering will always involves compromise, as steeper transitions and minimal ripple may seem ideal, but real-world signals can violate the assumptions of ideal filter theory, and the severity of phase smearing, ringing, and the suppression of desired components increases as one pushes spectral selectivity [18,19,22,51]. Consequently, filter choice should be guided by the signal characteristics and analysis goals, rather than by theoretical sharpness or smoothness alone [18,22,51]. In the present benchmark, all reported empirical statistics were computed using forward–backward (zero-phase) filtering to avoid phase-delay confounds in offline data. Causal SOS implementations were relevant for real-time systems (e.g., closed-loop devices or robotics) but were not used to generate the results reported here.

Gait acceleration resided in a low-frequency band. In past sensor gait analyses work, it had been noted that gait acceleration signals have low-frequency content of about 0.5–15Hz and small amplitude due to the biomechanics of human walking [12,52,53]. Wearable sensors therefore aimed to retain these slow components and suppress higher-frequency artifacts from strap vibration and sensor noise [12,52,54]. Reviews of IMU-based gait analyses noted that most studies employed low-pass filtering to eliminate high-frequency disturbances and that Butterworth filters were by far the most frequently used designs [12,53,55,56]. Crenna and colleagues further emphasized that zero-phase Butterworth low-pass filters with forward and reverse applications were among the most common filters in biomechanics precisely because they combined flat passbands with acceptable slopes [12,53,56]. This standard practice can smooth the signal while preserving waveform morphology and has been widely used for the double integration of acceleration to derive velocity and position [12,54,56]. Our results endorsed that choice as Butterworth filtering shared the SNR crown. Even so, the Reza filter edged ahead by preserving extra stride power within the 0.5–5 Hz gait band. In contrast, Chebyshev and elliptic filters, with their steep slopes and passband ripple, shaved away genuine motion content. When clinicians quantify stride-to-stride variability, preservation of the motion-related kinematic signal (e.g., acceleration time series) matters [12,52,54]. The Reza filter’s brick-wall profile can weed out chatter without clipping cadence, suggesting a promising balance between noise suppression and fidelity. However, as with any steep cutoff, parameters should match the features of interest (overly aggressive cutoffs can attenuate meaningful higher-frequency components), and standard padding/tapering at segment boundaries is recommended to minimize edge effects when filtering finite-length records.

Classical IIR designs were rooted in polynomial approximations and can inherit well-known trade-offs: Butterworth filters delivered a maximally flat passband but had relatively slow roll-off [28,30,51]; Chebyshev Type I filters sharpened the transition band by allowing equiripple gain in the passband, trading ripple for steeper skirts [28,30,51]; and elliptic (Cauer) filters permitted ripple in both passband and stopband to achieve the narrowest transition region for a given order, but at the cost of highly nonlinear phase and greater waveform distortion [28,30,51]. The Reza filter broke from this lineage by using an exponential window. Comparative studies into window functions showed that the exponential window provided the highest sidelobe roll-off ratio and maximum far-end stopband attenuation, outperforming Kaiser and Cosh windows [37]. In practice, this yielded a near-vertical cutoff with negligible ripple, providing a flat passband and deep stopband [30,37,51]; additionally, running the filter forward–backward (zero phase) eliminated phase distortion [12,54,56]. In our data, the Reza design achieved near-elliptic attenuation without introducing transient ringing, preserving waveform morphology while rejecting noise.

Our cohort comprised healthy young adults walking at comfortable speeds; consequently, these findings may not generalize to clinical populations with erratic gait or tremor where high-frequency bursts may carry important diagnostic information [12,52,54]. We also averaged across channels for simplicity, meaning axis-specific differences could reveal additional nuances in future work. A key next step is to test whether the filter-dependent differences observed here translate into downstream performance (e.g., feature stability, cross-subject generalization, and decoding accuracy in BCI/affective pipelines) which was beyond the scope of this signal-quality benchmark. In particular, research on muscle–computer interfaces [57], neuro-prosthetic control [58], and AIoT-based human activity recognition [59] could help identify the potential expanded utility of the Reza filter in future research efforts. Finally, the Reza filter is new, so researchers will need guidance on parameter tuning and boundary conditions, and its use should be guided by the specific analysis goal rather than replacing existing filters by default. Trials on pathological gait, electromyography, and EEG analyses that depend on phase relationships (e.g., phase coupling or cross-frequency coupling) are necessary to sharpen external validity, particularly to evaluate potential edge-related ringing effects near steep cutoffs (e.g., in delta and gamma ranges). Future work should include explicit time domain diagnostics (e.g., impulse/step responses, settling time, and ERP-aligned waveform checks) to quantify ringing and temporal smearing risks under steep cutoffs.

When analyses can hinge on delicate features (e.g., an ankle torque blip or a fleeting theta burst), it is worth revisiting the filter toolbox [18,22,51]. The Reza filter blends Butterworth-style fidelity with elliptic-grade selectivity, broadening the space where “clean” and “true” can coexist. No filter is a panacea as context rules, and investigators must decide how much ripple, phase shift, and computational complexity they can tolerate. As de Cheveigné & Nelken have warned, filters can smear temporal features and suppress genuine components, so simulations and careful parameter selection are essential [18,19,22,51]. Ultimately, noise should be a solvable nuisance, not the Achilles’ heel of movement science.

## 5. Conclusions

For EEG, SNR values were negative across filters (consistent with noise power exceeding in-band signal power under the adopted definition), and Chebyshev and elliptic yielded the highest (least negative) SNR, while Reza yielded the lowest SNR; meanwhile, the total 0.5–50 Hz band power did not differ by filter, even though Chebyshev Type I achieved the highest numerical SNR in our comparisons (consistent with prior cautions that SNR alone can be a misleading quality metric in electrophysiology; [19,23]). For IMU-based gait metrics, the Reza filter delivered Butterworth-level denoising while preserving more gait-band power, a desirable combination when estimating subtle stride-to-stride variability or fractal gait signatures. Still, no single filter is optimal for every study. Instead of defaulting to century-old designs, investigators should match ripple tolerance, phase-linearity requirements, and roll-off steepness to the specific scientific question and outcome variables at hand.

## Data Availability

The human subjects data used in this analysis are available via the public dataset published by Hanada, Kalabic, and Ferris (2024) [41]. The full Reza-Filter repository (including high-pass, low-pass, and band-pass implementations, example scripts, and analysis utilities) is available at: https://github.com/Rezapousti/Reza-Filter.git, accessed on 9 March 2026.

## Supplementary Materials

The full Reza-Filter repository (including high-pass, low-pass, and band-pass implementations, example scripts, and analysis utilities) is available at: https://github.com/Rezapousti/Reza-Filter.git.
